# Is all-inside with suspensory cortical button fixation a superior technique for anterior cruciate ligament reconstruction surgery? A systematic review and meta-analysis

**DOI:** 10.1186/s12891-020-03471-3

**Published:** 2020-07-07

**Authors:** Chun-Wei Fu, Wei-Cheng Chen, Yung-Chang Lu

**Affiliations:** Department of Orthopedic Surgery, Mackay Memorial Hospital, No. 92, Sec. 2, Zhongshan N. Rd., Zhongshan Dist., Taipei City, 104 Taiwan, R. O. C.

**Keywords:** Anterior cruciate ligament, ACL reconstruction, All-inside, Suspensory fixation, Interference screws

## Abstract

**Background:**

To compare the clinical results of all-inside anterior cruciate ligament reconstruction (ACLR) using suspensory cortical button fixation and full tibial tunnel drilling.

**Methods:**

Systematic searches were conducted of published literature up to November 2019 on PubMed, Embase, and Cochrane for studies comparing all-inside ACLR using suspensory cortical button fixation and full tibial tunnel ACLR. Two reviewers independently determined eligibility, extracted the outcome data, and assessed the risk of bias of the eligible studies. The clinical outcome and graft reruptures were pooled by using random effects with mean differences and risk ratios for continuous and dichotomous variables, respectively.

**Result:**

A total of nine studies (five randomized controlled trials and four comparative studies) involving 613 patients were included in the meta-analysis. The postoperative functional outcome, knee laxity measured with arthrometer, and graft reruptures were comparable between patients with all-inside ACLR using suspensory cortical button fixation and full tibial tunnel ACLR. However, a significantly greater thickness of autologous tendon was used and less change in drilling tunnel diameter was noted in patients with suspensory cortical button graft fixation.

**Conclusions:**

All-inside ACLR with suspensory cortical button fixation was not clinically superior to full tibial tunnel ACLR with interference screw fixation in functional outcomes, knee laxity measured with arthrometer, or rerupture rate. However, the advantage of using suspensory cortical button fixation was that a thicker graft could be used for reconstruction, and brought less tibia tunnel widening compared with bioabsorbable interference screw fixation.

## Background

**Fig. 1:**
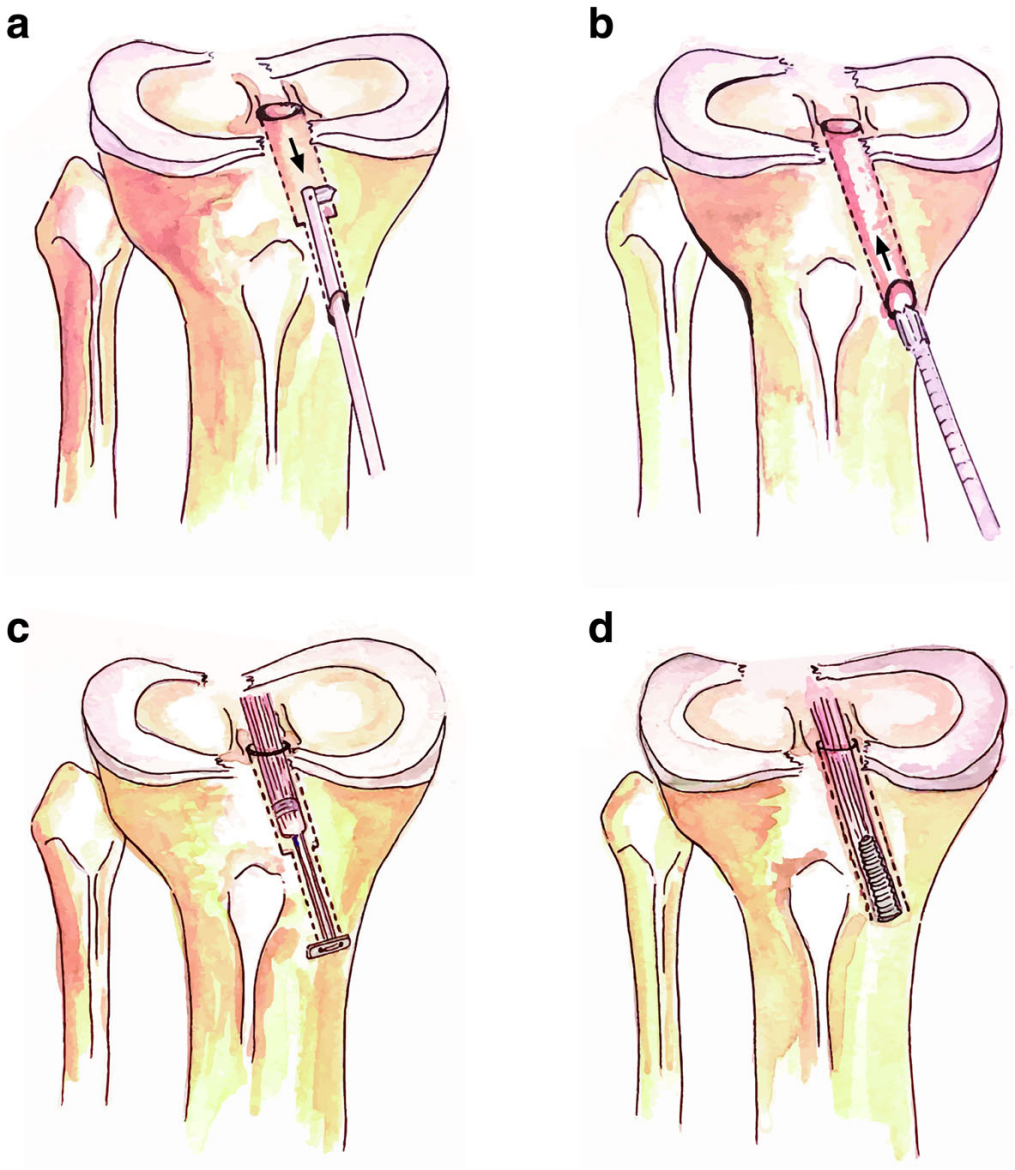
The figure illustrates preparation of the tibial tunnel method with retrograde drilling in all-inside ACLR (**a**) and conventional full tibial tunnel drilling (**b**). Tibial side graft fixation with suspensory cortical button (**c**) and with bioabsorbable interference screw (**d**). Note: Bone tunnel is depicted with a dotted line, and the arrows indicate the drilling direction

The all-inside technique of anterior cruciate ligament reconstruction (ACLR) is defined as creating the bone socket from the articular side of the tibia rather than conventional full-length tunneling through the knee joint and outer cortex [1]. With the technique evolved, the suspensory cortical button is mainly utilized as graft fixation method of all-inside technique (Fig. 1). The reported advantage of combining the all-inside technique and suspensory graft fixation includes biomechanically higher graft durability, greater preservation of flexion strength, and less bone tunnel widening in further follow-up [2–4]. However, whether the all-inside technique is superior to conventional full tibial tunnel ACLR remains controversial. A previous systematic review reported the satisfactory clinical outcome of all-inside ACLR, but few have described its comparison with the full tibial tunnel method [5]. The present systematic review and meta-analysis aimed to compare surgical outcomes between all-inside ACLR using suspensory cortical button fixation with full tibial tunnel ACLR with regard to function, knee stability, graft failure, and further bone tunnel widening.

## Methods

This is a systematic review of clinical outcome comparisons between ACLR using the all-inside technique with suspensory cortical button fixation compared with the full tibial tunnel method (with interference screw fixation). The Preferred Reporting Items for Systematic Reviews and Meta-Analyses guidelines and algorithm were used for this systematic review.

### Literature search and study selection

We performed the literature search on November 7, 2019 in the PubMed, Embase, and Cochrane databases, using the keywords “(Anterior cruciate ligament or ACL) and (All-inside, suture button, cortical button, or suspensory),” without a limitation for year of publication. The search was not restricted to randomized controlled trials due to the anticipated scarcity of published literature. Eligibility criteria for review inclusion were comparison studies of the all-inside ACLR technique with suspensory cortical button fixation and the full tibial tunnel ACLR technique, which use interference screw fixation on both the femur and tibia side or only on the tibia side. Cadaver or animal studies, biomechanical studies, literature reviews, and publication types unlikely to contain relevant information (news, comments, letters to the editor, and editorials) were excluded. Two independent reviewers (Chen and Fu) evaluated the eligibility of the selected studies. When necessary, we obtained full-text articles to determine eligibility for inclusion. Disagreements were resolved by discussion.

### Assessment of methodological quality

The methodological quality of the enrolled studies was evaluated by two reviewers independently, using Jadad scoring for the randomized controlled trials (RCTs) and the Newcastle–Ottawa Quality Assessment Scale for the nonrandomized comparative trials. The Jadad score evaluates RCT methodology according to three aspects: randomization (2 points), blinding (2 points), and an account of all patients (1 point). The range of potential scores is 0 to 5; a higher score indicates better methodological quality [6]. The Newcastle–Ottawa Quality Assessment Scale contains eight items in three categories: participant selection (four items), comparability (one item), and exposure (three items). A study can be scored a maximum of one point for items in the Selection and Exposure domains and a maximum of two points for the comparability domain [7].

### Data extraction

All the relevant data were extracted from the selected studies by two independent reviewers. Any disagreement or data inconsistency were resolved by discussion. Information about the first author, year of publication, study design, type of treatment arm, number of patients enrolled, mean age, follow-up time, graft type and thickness, fixation technique, and material used are shown in Table 1.

**Table 1:** Characteristic of included trials

Study name	Period	Study design, Level of evidence	No. of patients		Age		Follow-up time	Quality assessment
			All- inside	Full tibial tunnel	All- inside	Full tibial tunnel		
All-inside VS full tibial tunnel								
Desai et al., 2019 [8]	July 2011 to July 2015	Cohort study; Level 3.	82	54	25.8 ± 10.2	21.1 ± 7.3	All-inside: 30.1 mons, Full tibial tunnel: 25.8 mons	^b^8
Kouloumentas et al., 2019 [3]	2015 to 2016	RCT; level 1	45	45	27.6 ± 11.4	29.7 ± 11.0	24 mons	^a^5
Mayr et al., 2019 [9]	2013 to 2016	RCT; Level 2	17	16	26 ± 6	29 ± 7	24 mons	^a^2
Monaco et al., 2018 [10]	Jan 2016 to June 2016	Cohort study; Level 3	22	22	32.5 ± 6.7	31.7 ± 7.1	24 mons	^b^8
Baldassarri et al., 2018 [11]	Nov 2012 to Sep 2013	RCT; Level 2	28	31	24.7	25.2	48 mons	^a^2
Volpi et al., 2014 [12]	2007 to 2008	Cohort study; Level 3	20	20	38.4 ± 10.8	32.6 ± 9.3	24 mons	^b^5
Benea et al., 2013 [13]	Dec 2010 to Sep 2011	RCT; Level 1	22	22	28.4 ± 8.6	30.2 ± 9.4	6 mons	^a^3
Suspensory cortical button fixation (suspensory fixation) VS Resorbable interference screw fixation (interference screw)								
			Suspensory fixation	Interference screw	Suspensory fixation	Interference screw		
^c^Colombet et al., 2016 [14]	Feb 2014 to Sep 2014	Prospective cohort study; level 2	60	49	28.9 ± 9.5	27.6 ± 6.8	6.6 mons	^b^8
^c^Lubowitz et al., 2015 [15]	NA	RCT; Level 1	31	27	40.2 ± 11.9	41.6 ± 9.1	24 mons	^a^3

*ST4* Quadrupled semitendinosus tendon, *DGST* Doubled gracilis and semitendinosus tendons, *RCT* Randomized controlled trial

^a^indicates that the study was evaluated using Jadad’s scale

^b^indicates that the study was evaluated by Newcastle–Ottawa scale

^c^Two studies (Colombet, 2016 and Lubowitz, 2015) that partially met the inclusion criteria comparing suspensory cortical button fixation and interference screw fixation were included for tunnel widening analysis

The outcome measures, functional outcomes (Lysholm score, subjective and objective International Knee Documentation Committee [IKDC] score, Tegner activity scale, and Knee Society Score [KSS]), knee stability (measured using the KT-1000 instrumented knee laxity device), change of bone tunnel width, and tendon rerupture were extracted and analyzed.

### Statistical analysis

In the outcome data analysis, we synthesized the continuous outcome data by using the mean difference and standard deviation (SD). If only a range was reported, the estimated SD was calculated by range/4 in moderately sized samples (15 < *n* ≤ 70) and by range/6 in large samples (*n* > 70) [16]. For the objective IKDC score evaluation, the extracted data were added and a chi-squared test with Fisher’s correction was used for between-group difference analyses. The standardized mean differences (SMDs) of the extracted data were indicated to represent a favorable treatment option. For dichotomous outcome data, we used the odds ratio (OR) for synthesis. A random-effects model was used to pool individual SMDs and ORs. The 95% confidence intervals (CI) were calculated for each outcome. Between-trial heterogeneity was determined by performing the I^2^ test; values > 50% were regarded as indicating considerable heterogeneity. All analyses were performed using Comprehensive Meta-Analysis software (Version 3.3.070).

## Results

### Study selection and critical appraisal

**Fig. 2:**
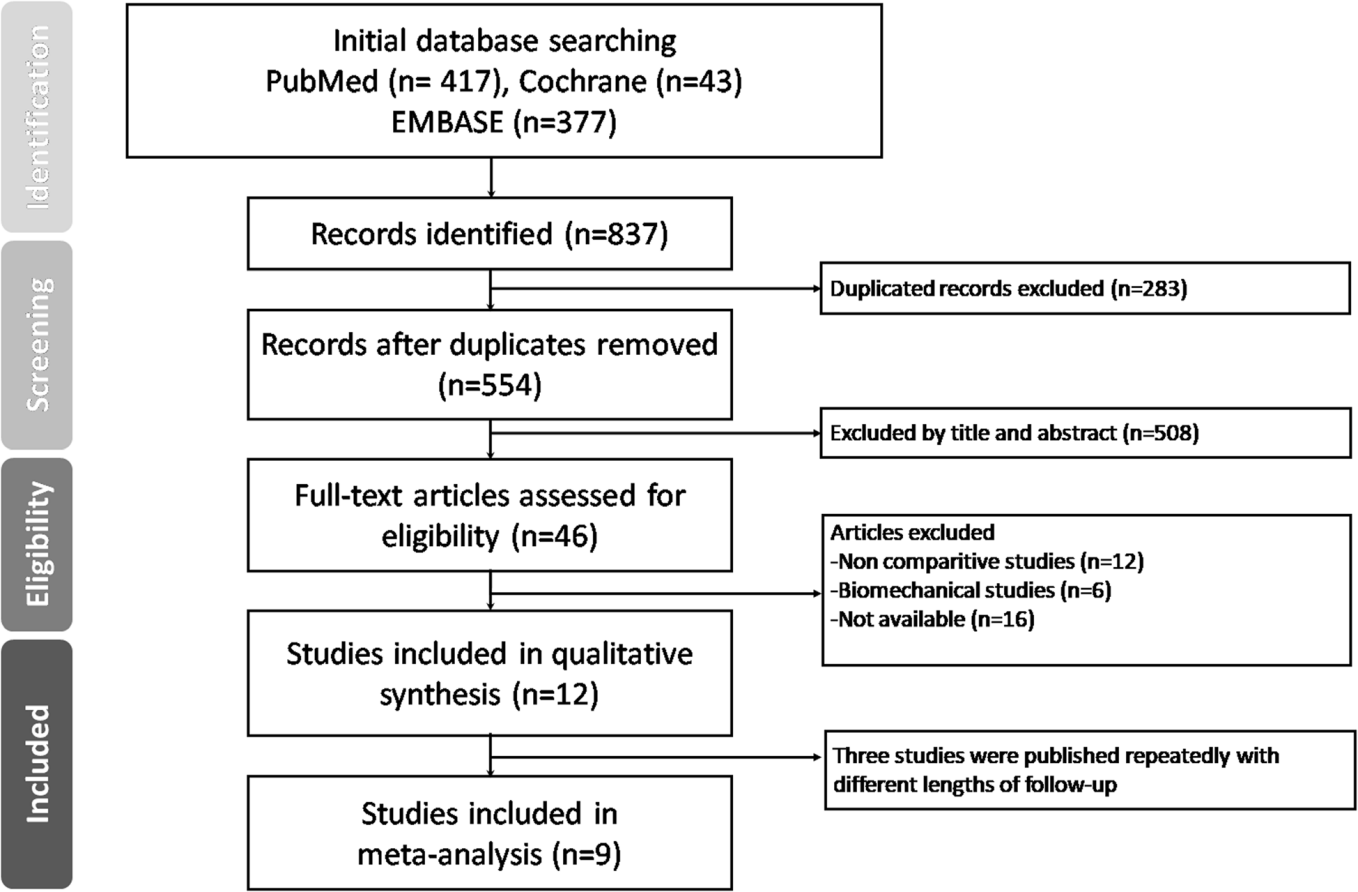
Preferred reporting items for systematic reviews and meta-analysis (PRISMA) flow diagram for the searching and identification of included studies

The study’s search criteria, exclusion criteria, and final selection of studies are presented in a flow diagram from the Preferred Reporting Items for Systemic Reviews and Meta-Analysis (PRISMA) guidelines (Fig. 2). Three studies [4, 17, 18] were published repeatedly with different lengths of follow-up; thus, we extracted the data from the most recent of them. We did not include Lubowitz’s RCTs published in 2013 comparing outcomes between the all-inside and full tibial tunnel ACLR methods, with both groups using aperture fixation on the femur and tibia side instead of the suspensory cortical button fixation device [19]. However, in order to reveal whether the fixation device was associated with widening of the drill tunnel, we included two studies that partially met the inclusion criteria for analysis. The first study is Lubowitz’s RCT published in 2015, comparing the outcome between adjustable suspensory cortical button fixation and aperture fixation, with both groups using the all-inside drilling technique; we extracted only the data regarding the postoperative follow-up tunnel diameter [15]. The second study is Colombet’s prospective cohort study published in 2016 comparing tunnel diameter changes between patients who had undergone full tibial tunnel ACLR using suspensory cortical button fixation or bioabsorbable screw fixation, and we collected the tunnel diameter change data for further analysis [14]. We included 5 RCTs and four comparative cohort studies published between 2013 and 2019 for the final meta-analysis. All the selected studies compared the all-inside ACLR technique (both femoral and tibial side bone socket) to the full tibial tunnel ACLR approach (femoral socket and full-length transtibial tunnel). The methodological quality of the RCTs was evaluated with the Jadad score. One study [3], clearly mentioning the method for randomization, appropriate blinding, and the withdrawal of patients from follow-up, scored five points. Two studies [13, 15] scored 3 points with all randomization, blinding, and withdrawals documented; however, the detailed method of randomization and blinding was not mentioned. Two studies [11, 17] that only mentioned the randomization and withdrawals scored 2 points. The three comparative studies were measured using the Newcastle–Ottawa scale. The study characteristics are presented in Table 1. Various outcome measures, detailed graft type, and fixation materials between the studies are listed in Table 2.

**Table 2:** Outcome measurement, graft type, and fixation materials of the studies

Author, year	Outcome measurement	Graft type/Graft thickness		Fixation material	
		All-inside	Full tibial tunnel	All-inside	Full tibial tunnel
All-inside VS full tibial tunnel					
Desai et al., 2019 [8]	Lachman test, pivot shift, Tegner activity scale, Lysholm score, IKDC score, and complications (includes graft failure)	ST4 (74.4%), 9.0 mm (range, 8.0–10.5 mm; SD, 0.6 mm)	DGST, 8.3 mm (range, 7.0–10.0 mm; SD,–0.7 mm)	Femoral side: TightRope (Arthrex), Tibial side: GraftLink (Arthrex)	Femoral side: Endobutton (Smith &Nephew)(65%), TightRope (Arthrex)(34%), or RetroButton (Arthrex)(1%) Tibial side: Interference screw Bio-Compression Screw (Arthrex)
Kouloumentas et al., 2019 [3]	Lysholm score, IKDC score, KOOS, KSS, knee laxity assessment (use KT-1000 arthrometer), isokinetic testing, and graft failure	ST4, Femoral side:8.2 ± 0.7 mm Tibial side: 8.3 ± 5.0 mm	DGST, Femoral side: 7.7 ± 0.5 mm Tibial side: 7.7 ± 4.9 mm	Both femoral and tibial side: TightRope (Arthrex)	Femoral side: Flipptack™ button system (Karl Storz, Tuttlingen) Tibial side: interference screw - Megafix (Karl Storz, Tuttlingen).
Mayr et al., 2019 [9]	Pivot shift, Tegner activity scores, Lysholm score, IKDC score, knee laxity assessment (use KT-1000 arthrometer), hop testing, and tunnel diameter and volume measured with CT scans	ST4, Femoral side: 7.7 ± 0.8 mm Tibial side: 8.0 ± 0.5 mm	DGST, Femoral side: 7.3 ± 0.5 mm Tibial side: 7.9 ± 0.8 mm	Both femoral and tibial side: TightRope (Arthrex)	Both femoral and tibial side: Interference screw-BioComposite (Arthrex)
Monaco et al., 2018 [10]	Tegner activity score, Lysholm scores, IKDC score, KSS, knee laxity assessment (use KT-1000 arthrometer), and tunnel diameter measured with CT scans	ST4	DGST	Both femoral and tibial side: TightRope (Arthrex)	Femoral side: TightRope (Arthrex) Tibial side: interference screw- Deltascrew (Arthrex)
Baldassarri et al., 2018 [11]	Marx score, Tegner activity score, IKDC score, and return to sport	NA	NA	Both femoral and tibial side: suspensory cortical buttons	Both femoral and tibial side: Interference screw
Volpi et al., 2014 [12]	Tegner activity score, Lysholm score, IKDC score, VAS	ST4	DGST	Both femoral and tibial side: suspensory cortical buttons	Femoral side: Interference screw or cortical suture button Tibial side: interference screw
Benea et al., 2013 [13]	IKDC score, VAS, knee laxity assessment (use Rollimeter arthrometer), tunnel position measured with X-ray	ST4	DGST	Femoral side: Tightrope (Arthrex) Tibial side: SutureButton (Arthrex)	Femoral side: Interference screw Tibial side: Interference screw
Suspensory cortical button fixation (suspensory fixation) VS Resorbable interference screw fixation (interference screw)					
		Suspensory fixation	Interference screw	Suspensory fixation	Interference screw
^a^Colombet et al., 2016 [14]	knee laxity assessment (use GeNouRoB arthrometer), Graft and tunnel measurement with MRI	ST4	ST4	Both femoral and tibial side: PULLUP (Science & BioMaterials) suspensory system	Femoral side: PULLUP (Science & BioMaterials) suspensory system Tibial side: Interference screw (LIGAFIX 60 (Science & BioMaterials))
^a^Lubowitz et al., 2015 [15]	IKDC score, KSS, SF-12 score, VAS, narcotic consumption, knee laxity assessment (use KT-1000 arthrometer), and tunnel diameter measured with X-ray	2-strand tibialis posterior tendon	2-strand tibialis posterior tendon	Femoral side: RetroButton (Arthrex) Tibial side: Titanium cortical button (Arthrex)	Femoral side: Interference screw-BioComposite (Arthrex) Tibial side: interference screw- RetroScrew (Arthrex)

*ST4* Quadrupled semitendinosus tendon, *DGST* Doubled gracilis and semitendinosus tendons, *IKDC* International Knee Documentation Committee, *KOSS* Knee Injury and Osteoarthritis Score, *KSS* Knee Society Score, *CT* Computed tomography, *VAS* Visual analog score, *SF-12* Short Form 12, *MRI* Magnetic resonance imaging

^a^Two studies (Colombet, 2016 and Lubowitz, 2015) partially met the inclusion criteria comparing suspensory cortical button fixation and interference screw fixation were included for the tunnel widening analysis

### Results of individual studies

#### Graft harvest and fixation device

In autologous graft selection, six studies [3, 8–10, 12, 13] used quadrupled semitendinosus tendon (ST4) in the all-inside group and double gracilis and semitendinosus tendon (DGST) in the full tibial tunnel group; one study did not clearly mention the autologous tendon graft donor site [11]. All the studies mentioned the suspensory cortical button graft fixation device was used on both the femoral and tibial side in the all-inside group of patients. However, for the full tibial tunnel ACLR groups, three studies [3, 8, 10] used femoral side cortical button fixation with tibial side interference screw fixation, and the other four studies mentioned interference screw graft fixation on both sides. The details of fixation device choice are shown in Table 1.

#### Graft size and flexion strength

**Fig. 3:**
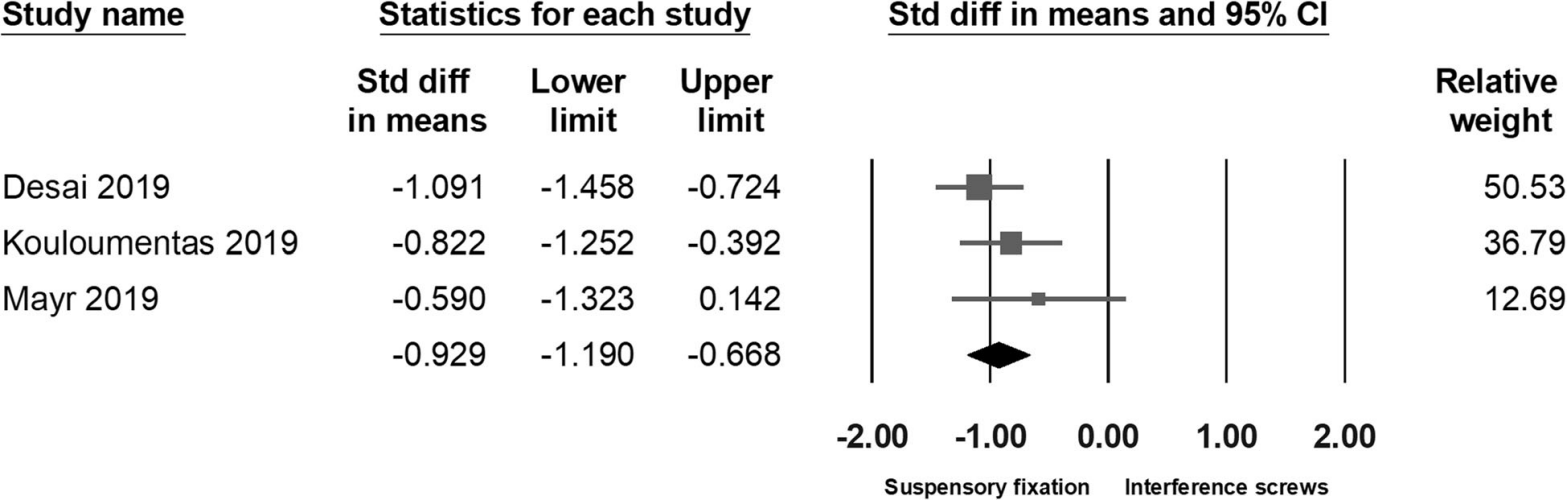
Forest plot of the graft size, Random, Heterogeneity: Tau^2^ = 0.000; Chi^2^ = 1.809, df = 2 (*p* = 0.405); I^2^ = 0%. Test for overall effect: Z = − 6.975 (*p* < 0.001)

Given quadrupled semitendinosus tendon is an inherent property of the all-inside with suspensory cortical button fixation technique, it is the only method that could achieve adequate graft length and thickness. Three studies had documented the autograft thickness. Desai et al. and Kouloumentas et al. had reported that graft size in the all-inside group (mostly using ST4) was significantly thicker than in the full tibial tunnel group (mostly use DGST); a similar result was also noted in our pooled data (95% CI − 1.190 to − 0.668; *p* < 0.001) (Fig. 3).

Flexion strength was investigated by Kouloumentas et al. and Monaco et al. Both these studies stated that better flexion strength was noted in the group of patients treated with all-inside ACLR [3, 4]. Although the data from the two studies could not be pooled due to different evaluation methods of flexion strength, the studies stated that preservation of the gracilis tendon might be associated with minor donor site morbidity and better flexion strength recovery.

### Functional outcomes

The studies investigated the functional outcome with several types of parameters at various times. We have extracted the available data on the last clinical follow-up, and the following score measurements include Lysholm score, subjective and objective IKDC, Tegner activity score, and KSS.

### Lysholm score

**Fig. 4:**
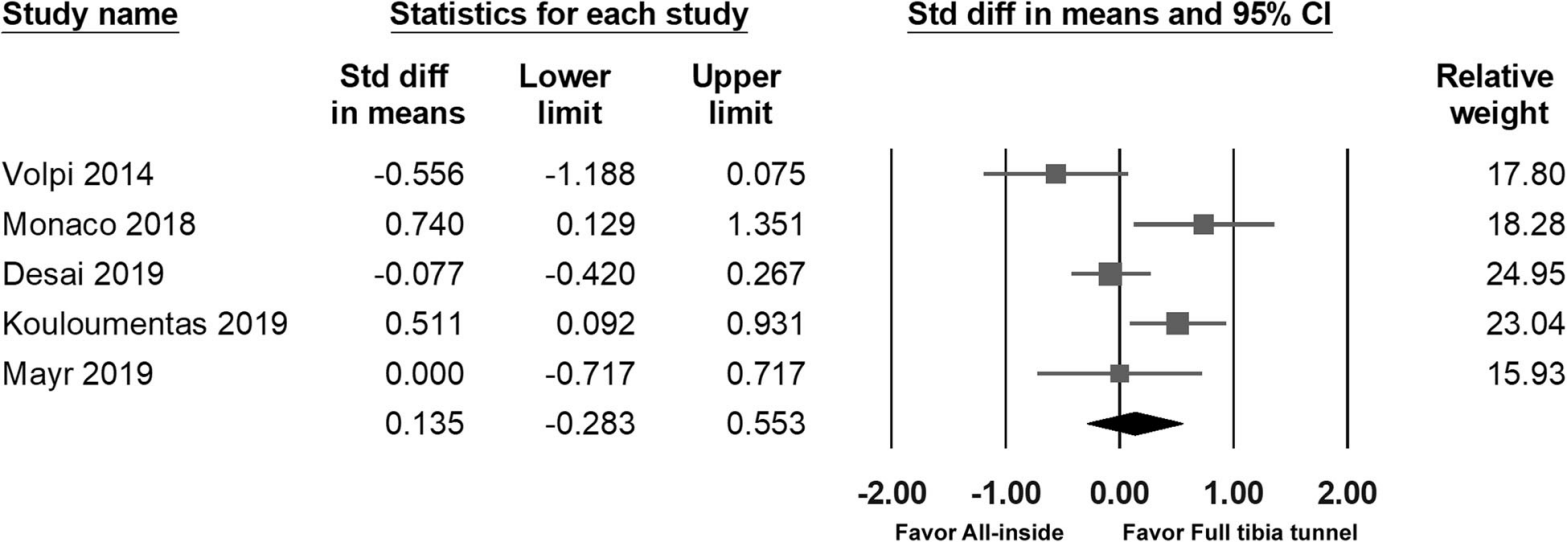
Forest plot of Lysholm score, Random, Heterogeneity: Tau^2^ = 0.151; Chi^2^ = 13.051, df = 4 (*p* = 0.011); I^2^ = 69%. Test for overall effect: Z = 0.634 (*p* = 0.526)

Five studies measured the Lysholm score; no significant differences were found between the two groups. The pooled data also found no significant between-group differences (95% CI − 0.283 to − 0.553; *p* = 0.526) (Fig. 4).

### Subjective and objective IKDC score

**Fig. 5:**
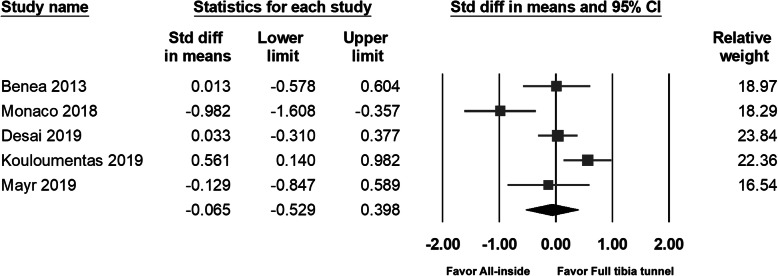
Forest plot of subjective IKDC score, Random, Heterogeneity: Tau^2^ = 0.204; Chi^2^ = 16.358, df = 4 (*p* = 0.003); I^2^ = 76%. Test for overall effect: Z = − 0.276 (*p* = 0.783)

Both the subjective and objective IKDC scores were measured by five studies. Significant postoperative improvement was noted in both the all-inside and full tibial tunnel groups; however, no significant between-group difference was found in postoperative score measurement. The pooled data of the subjective IKDC found no significant between-group differences (95% CI − 0.283 to 0.553; *p* = 0.526) (Fig. 5). Comparison of the postoperative objective IKDC scores also showed no significant differences (*p* = 0.189) (Table 3).

**Table 3:** Postoperative objective IKDC score

	All- inside group	Full tibial tunnel	*P*-value
IKDC A	86	93	0.189
IKDC B	35	33	
IKDC C	9	3	
IKDC D	0	0	

Note: Variables are expressed as *n*; IKDC, International Knee Documentation Committee

### Tegner activity score

**Fig. 6:**
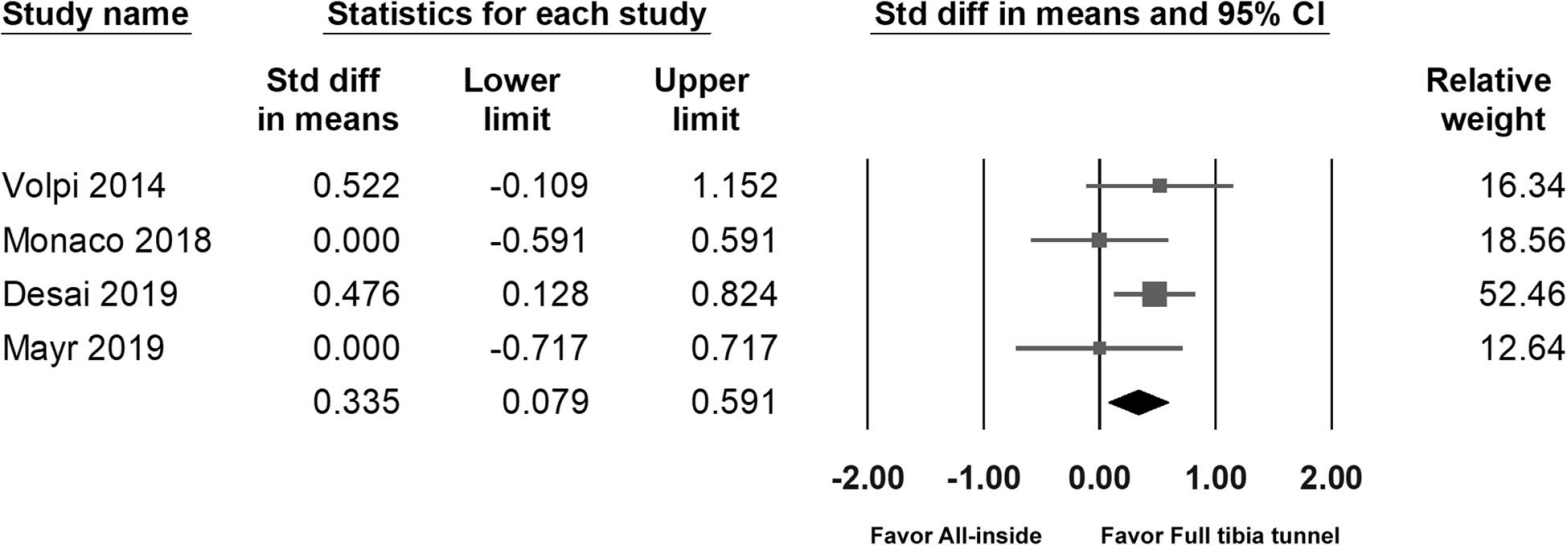
Forest plot of Tegner score, Random, Heterogeneity: Tau^2^ = 0.001; Chi^2^ = 3.307, df = 3 (*p* = 0.386); I^2^ = 1%. Test for overall effect: Z = 2.564 (*p* = 0.01)

The Tegner activity score data were extracted from four studies. A significantly higher Tegner activity score in the full tibial tunnel group (6.4 VS 6.8, *p* = 0.48) was noted in Desai’s study. The pooled data also showed a significantly higher score in the full tibial tunnel group (95% CI 0.079 to 0.591; *p* = 0.01) (Fig. 6). However, Desai et al. had stated that both groups of patients could reach the preinjury level of activity (preinjury score, 6.6 in the all-inside group and 7.0 in the full tibial tunnel group); thus, the between-group difference was not clinically significant.

### KSS

**Fig. 7:**
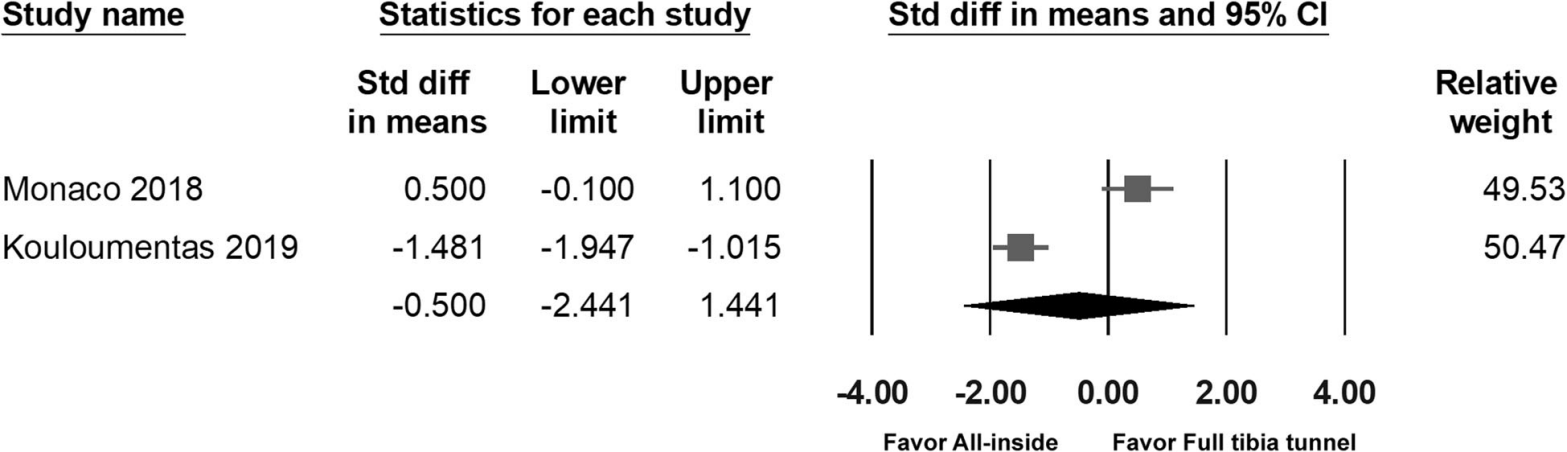
Forest plot of KSS, Random, Heterogeneity: Tau^2^ = 1.887; Chi^2^ = 26.095, df = 1 (*p* = 0.000); I^2^ = 96%. Test for overall effect: Z = -0.505 (*p* = 0.614)

Two studies measured the KSS. There was no significant difference between the groups in any study, or after pooling of the data (95% CI − 2.441 to 1.441; *p* = 0.614) (Fig. 7).

### Laxity measured by arthrometer

**Fig. 8:**
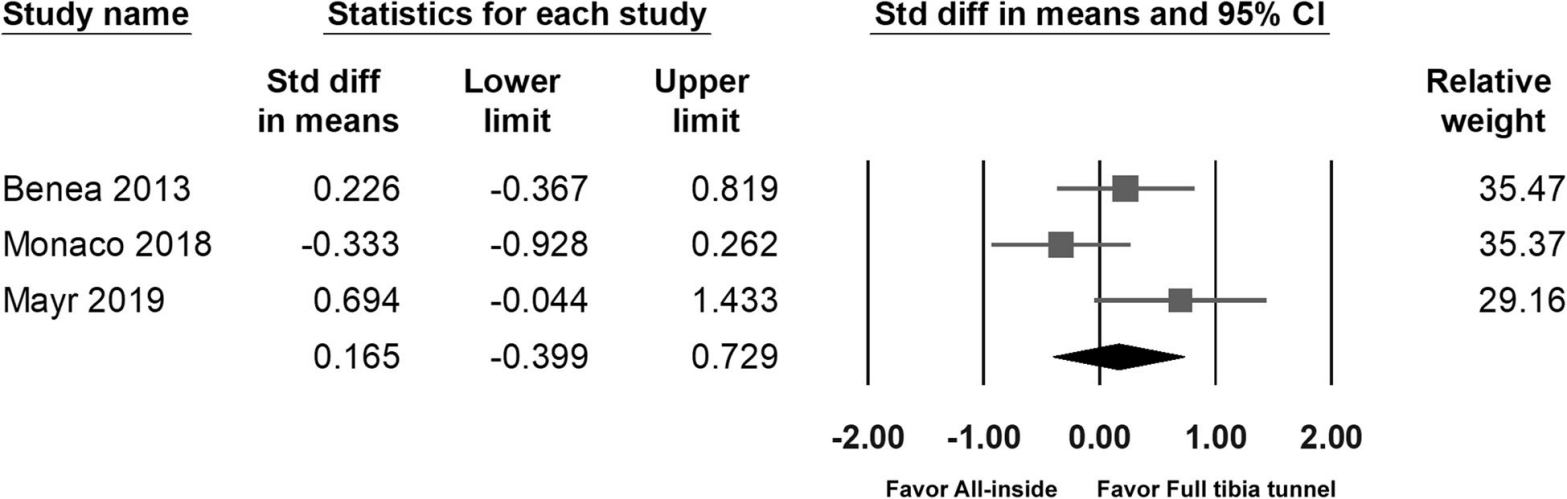
Forest plot of the knee laxity measured by arthrometer, Random, Heterogeneity: Tau^2^ = 0.142; Chi^2^ = 4.674, df = 2 (*p* = 0.097); I^2^ = 57%. Test for overall effect: Z = 0.573 (*p* = 0.567)

Two studies investigated the anteroposterior knee stability of the operative knee using the KT-1000 arthrometer (MedMetric Corporation, San Diego, CA, USA) [9, 10], and one study [13] used the Rolimeter (Aircast, Europe). All the studies stated that knee stability improved significantly postoperatively, but no significant difference between groups was noted. Given both the KT-1000 and Rolimeter provided a valid measure of knee laxity of the patients with ACL injury [20], we pooled the postoperative data measured using both types of arthrometer. We found that postoperative knee stability (95% CI − 0.399 to 0.729; *p* = 0.567) (Fig. 8) was comparable between the groups.

### Tunnel widening

**Fig. 9:**
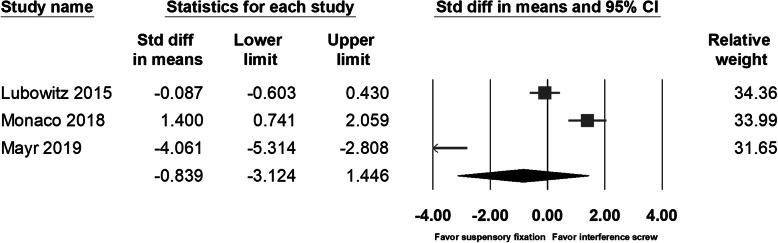
Forest plot of the direct postoperative tunnel width, Random, Heterogeneity: Tau^2^ = 3.885; Chi^2^ = 57.712, df = 2 (*p* = 0.000); I^2^ = 97%. Test for overall effect: Z = -0.720 (*p* = 0.472)

**Fig. 10:**
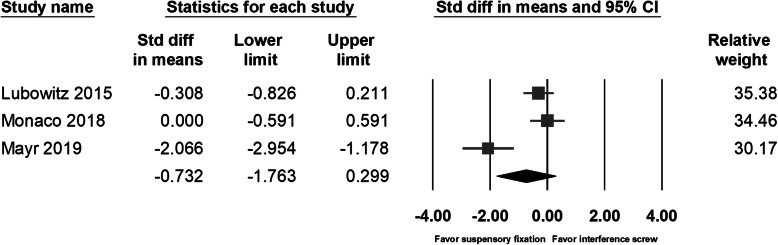
Forest plot of the follow-up tunnel width, Random, Heterogeneity: Tau^2^ = 0.712; Chi^2^ = 15.225, df = 2 (*p* = 0.000); I^2^ = 87%. Test for overall effect: Z = -1.392 (*p* = 0.164)

**Fig. 11:**
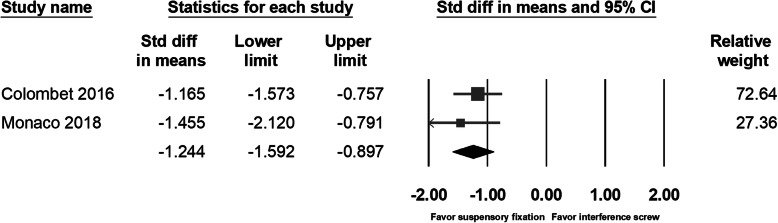
Forest plot of the tunnel diameter change, Random, Heterogeneity: Tau^2^ = 0.000; Chi^2^ = 0.533, df = 1 (*p* = 0.465); I^2^ = 0%. Test for overall effect: Z = -7.015 (p < 0.001)

The phenomenon of drill tunnel widening had been investigated by several studies [9, 10, 14, 15, 17] and was considered to be associated with not only the method of tunnel prepared but also by the graft fixation device. Thus, we included two more comparative studies investigating the difference between tunnel widening by suspensory cortical button fixation and by interference screws [14, 15]. Lubowitz et al. had found no between-group difference in tunnel diameter as measured with plain film radiography. In Mayr’s study [17], with computed tomography as a main tool for measurement, a significant femoral tunnel volume increase was noted in the all-inside group at 6 months’ follow-up; however, in the other study with longer follow-up times (24 months) for the same groups of patients, significantly increased tibia volumes and diameters were found in the full tibial tunnel group [9]. Studies by Monaco et al. and Colombet et al. had found significant tibia tunnel widening in the group with interference screw fixation. Due to the different type of imaging study for evaluating the diameter or volume of the drilling tunnel, we could only compile the data of tibia tunnel diameter from three studies. The pooled data showed no significant between-group differences in the direct postoperative tunnel width (95% CI − 3.124 to 1.446; *p* = 0.472) (Fig. 9) or the follow-up tunnel width (95% CI − 1.763 to 0.299; *p* = 0.164) (Fig. 10). However, when analyzing the tunnel diameter change, individual studies and the pooled data showed significantly increased tunnel diameter in the patients with interference screw fixation (95% CI − 1.592 to − 0.897; *p* < 0.001) (Fig. 11).

### Rerupture

**Fig. 12:**
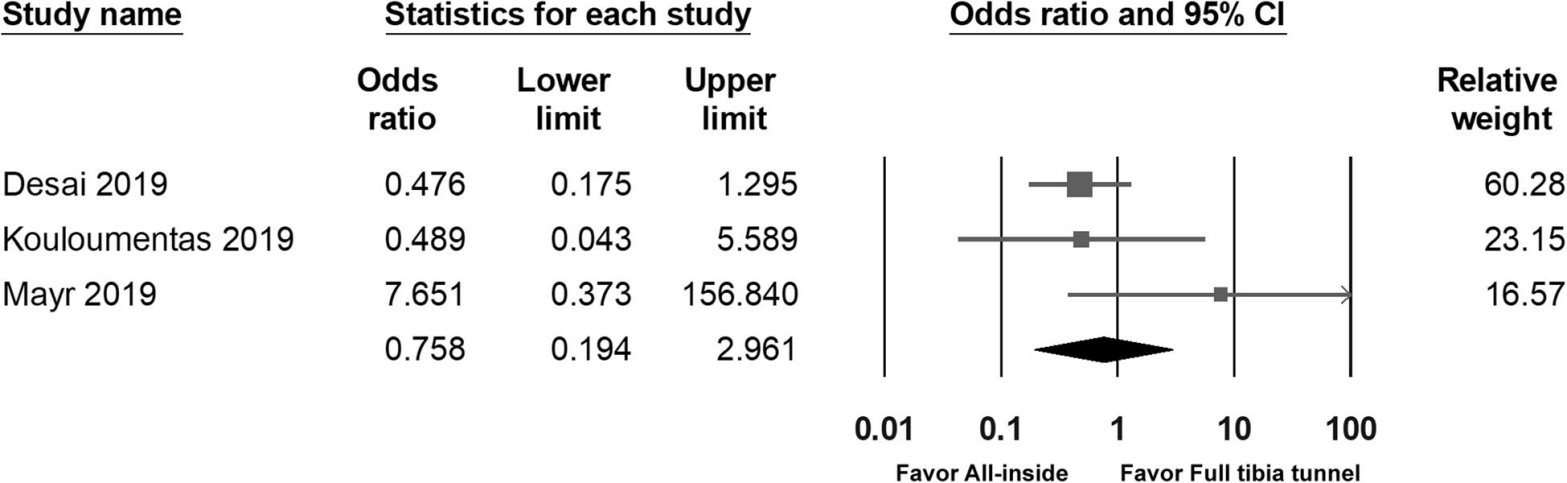
Forest plot of the re-rupture, Random, Heterogeneity: Tau^2^ = 0.540; Chi^2^ = 2962, df = 2 (*p* = 0.227); I^2^ = 32%. Test for overall effect: Z = -0.398 (*p* = 0.691)

Rerupture was described in three studies. There was no significant difference between groups within any study or after pooling of data (OR 0.758; 95% CI 0.194 to 2.961; *p* = 0.691) (Fig. 12).

## Discussion

The present meta-analysis focused on an outcome comparison between ACLR using the all-inside technique with suspensory cortical button graft fixation and ACLR using the full tibial tunnel technique. It included five RCTs and four retrospective cohort studies comparing these two types of surgical methods. With both femur and tibial side suspensory cortical button graft fixation use, the greater graft thickness of quadrupled semitendinosus tendon was available for ligament reconstruction. Less tibial tunnel widening in the further follow-up was observed in those patients with suspensory cortical button graft fixation. The present analysis showed that the all-inside ACLR technique was not critically superior to the full tibial tunnel technique in functional outcomes, knee laxity measured with arthrometer, or tendon rerupture rate.

A previous systematic review by de Sa et al. in 2018 had reported a low graft failure rate and ideal clinical improvement using the all-inside ACLR technique. However, the review focused on compiling the reported outcomes from individual studies rather than the comparison between different surgical methods [5].

Since the last review of de Sa et al., several studies have directly compared the all-inside ACLR with the full tibial tunnel technique. Thus, we hypothesized that an analysis of the comparison studies would provide some valuable insights into the ongoing debate. Browning et al. had systematically reviewed ACLR using suspensory or aperture fixation and found that the suspensory device engendered better knee stability and less graft failure [21]. Recently, with the evolution of surgical instruments and techniques, suspensory devices are used in most all-inside ACLRs. Thus, both the bone tunnel preparation technique and the fixation device might affect the clinical outcome of ACLR.

The difference in functional outcome between the two methods was not significant in the individual studies or in our pooled data. First, according to most of the studies, we noted that ACLR is a surgery with high patient satisfaction, and most patients felt clear improvement postoperatively. Second, the follow-up time in the selected studies was relatively short, which might not have been sufficient to reveal fixation failure because of the screw degradation process or the tunnel widening phenomenon [22]. In regard to postoperative pain, three studies had mentioned this outcome measurement. However, the data could not be pooled, given it was assessed in different ways and at different times. The individual studies reported comparable postoperative pain and analgesic consumption for both the all-inside and full tibial tunnel groups at all follow-up times [12, 13, 15]. Return to sports was investigated by two studies [8, 11]; however, the data were recorded in a different time frame. Baldassarri et al. stated that the patients who underwent full tibial tunnel ACLR showed slightly better performance in the postoperative 6–8 months’ follow-up, but this difference became insignificant in further follow-up. Desai et al. found that the mean time for return to sports was longer in the patients with all-inside ACLR (12.5 vs 9.9 months). Although the return to sports is an important parameter to measure graft maturation of the ACLR, it varies between the types of sports and rehabilitation protocols. More studies comparing the rate and level of return to sports are warranted.

With suspensory cortical button fixation, the necessity of harvested graft length was approximately 28 cm, and could be achieved with only semitendinosus tendon harvesting most of the time [23]. The previous systemic reviews stated that tendon graft thickness of 8 mm had lower failure rates in ACLR, and this lower limit diameter could also be achieved by using quadrupled semitendinosus tendon (ST4) graft in most cases, regarded as inherent property of the all-inside ACLR with suspensory cortical button fixation technique [24]. However, there are many factors that might influence the thickness of the tendon graft such as age, gender, and body height. Thus, it must be noted that every patient cannot not achieve the ideal graft length and thickness by using the semitendinosus tendon only, and the gracilis must be secondarily harvested.

Some studies have shown that this gracilis-sparing technique could achieve more minimal surgical incision and less donor site morbidity [1, 25]. Since both the semitendinosus and gracilis tendon function both as knee flexors and tibial internal rotators, harvest causes weakness of internal tibial rotation. Both Kouloumentus et al. and Monaco et al. had stated that the improved flexion strength in the group of patients who underwent all-inside ACLR could be attributed to the gracilis-sparing technique [3, 4], and it is beneficial to functional activity or sports with high demands on hamstring muscle strength [25].

Tunnel widening is always a concern in ACL reconstruction surgery. In the biomechanical aspect, synovial fluid penetration and micromovements at the graft to bone interface (bungee and windshield wiper effect) might enlarge the tunnel. The inflammatory response or foreign body reaction to the bioabsorbable screw could be the biological cause for tunnel widening. Using advanced imaging, the current studies have shown significant increases in tunnel volume in the group of patients with bioabsorbable interference screws or even cyst formation at follow-up [9, 14]. Although the current evidence could not prove a correlation between tunnel widening and poor clinical outcomes or graft failure, the concern is that bone loss of the tunnel might be an obstacle for revision surgery, given the graft failure rate in young athletes was high [26]. Further, we found that two of the studies [9, 15] measured the bone tunnel diameter of postoperative radiography as the reference point to determine the tunnel widening, however two of the studies [10, 14] use the initial drill diameter as the reference point. The inconsistency in tunnel measurement might have raised potential bias since the bone tunnel diameter could easily be altered by drilling or tightening the interference screw during the surgery. Thus, standardized volume measurement might help us shed light on the change in tunnel volume.

In our review, the pooled data showed that graft failure was similar between the all-inside and full tibial tunnel ACLR. However, the all-inside technique of ACLR exhibited a trend toward longer operation time [9, 15]. Furthermore, care must be taken with regard to surgical complications related to suspensory cortical button use, such as a dislodged button or suture breakage, as reported in a previous study. The selected studies reported that the study period included the surgeon’s learning curve on the newly developed all-inside technique. The unfamiliarity with the surgical instrument and fixation device might be the cause of all these complications [3, 8, 9, 12, 13]. Finally, the all-inside technique is dependent on the retrograde drilling technique, which requires specific surgical instruments, such as a retrograde reaming device and suspensory fixation device, which are sold by certain companies as listed in Table 2. The relation between these factors and the potential conflict of interests declared by the selected studies must be considered [8, 10, 13–15].

### Limitations

There are several limitations of the present meta-analysis. First, the quality of the available studies is low. The five RCTs and four comparative studies described only 613 patients, which is low, considering the high incidence of ACL injury. Variations in study design, patient characteristics, sample size, reporting of outcome, and postoperative protocol resulted in high heterogeneity between the studies. The identification of an anatomical landmark for tunnel positioning varied between surgeons and was rarely mentioned in these studies. Second, we did not serially investigate outcome measurement; instead, we used the data of the last follow-up, which were commonly documented to represent the final postoperative status. Besides, the follow-up period in the selected studies were short-term to mid-term (from 6 months to 48 months) (Table 1), which may raise a concern that some complications such as graft loosening, implant breakage, or revision surgery might occur after 5 years. More studies investigating the long-term follow-up were needed to prove the reliability of this new technique and implants. Third, although the bioabsorbable interference screw has been frequently used in ACLR [27, 28] and was selected as a control technique in our selected studies, other graft fixation methods are still available, such as metallic interference screw, cross-pin, and staple fixation, which have played roles in current ACLR surgery. However, there is a lack of evidence to compare these techniques or implant fixation to the all-inside technique, and thus, it is hard to determine the optimal method.

## Conclusion

In this systematic review and meta-analysis, with a limited follow-up period, we found that the all-inside ACLR technique with suspensory cortical button fixation was not clinically superior to the full tibial tunnel technique with interference screw fixation in functional outcome and knee laxity as measured with an arthrometer. However, the advantages of using suspensory cortical button fixation included the use of a thicker ST4 graft for reconstruction, and brought less tibia tunnel widening compared with bioabsorbable interference screw fixation.

## Supplementary information

**Additional file 1: Figure S1.** Funnel plot of the graft size.

**Additional file 2: Figure S2.** Funnel plot of Lysholm score.

**Additional file 3: Figure S3.** Funnel plot of subjective IKDC score.

**Additional file 4: Figure S4.** Funnel plot of Tegner score.

**Additional file 5: Figure S5.** Funnel plot of the knee laxity measured by arthrometer.

**Additional file 6: Figure S6.** Funnel plot of the direct postoperative tunnel width.

**Additional file 7: Figure S7.** Funnel plot of the follow-up tunnel width.

**Additional file 8: Figure S8.** Funnel plot of the re-rupture.

## Data Availability

Not applicable. The data used for analysis was retrieved from openly published studies listed in our manuscript.

## Abbreviations

ACLR: Anterior cruciate ligament reconstruction
RCTs: Randomized controlled trials
IKDC: International Knee Documentation Committee
KSS: Knee Society Score
SD: Standard deviation
SMDs: Standardized mean differences
OR: Odds ratio
CI: Confidence intervals
PRISMA: Preferred Reporting Items for Systemic Reviews and Meta-Analysis
ST4: Quadrupled semitendinosus tendon
DGST: Doubled gracilis and semitendinosus tendon
