# Supplementary figures and images for: Is all-inside with suspensory cortical button fixation a superior technique for anterior cruciate ligament reconstruction surgery? A systematic review and meta-analysis
Source: BMC Musculoskelet Disord. 2020 Jul 7;21:445. doi: 10.1186/s12891-020-03471-3 (PMC7341582; doi:10.1186/s12891-020-03471-3)

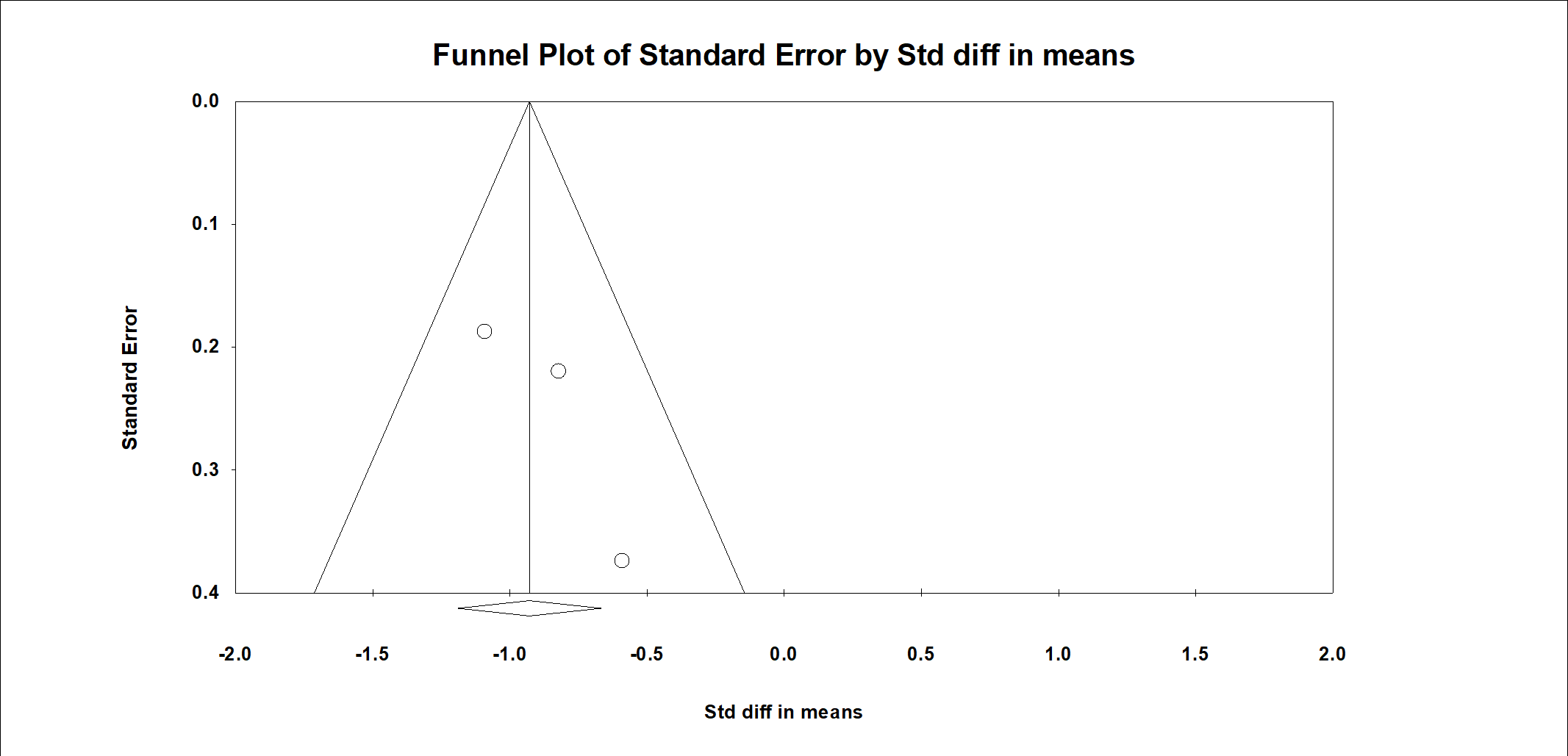

Supplement: Supplementary file 1 — Additional file 1: Figure S1. Funnel plot of the graft size. [file 12891_2020_3471_MOESM1_ESM.tif]

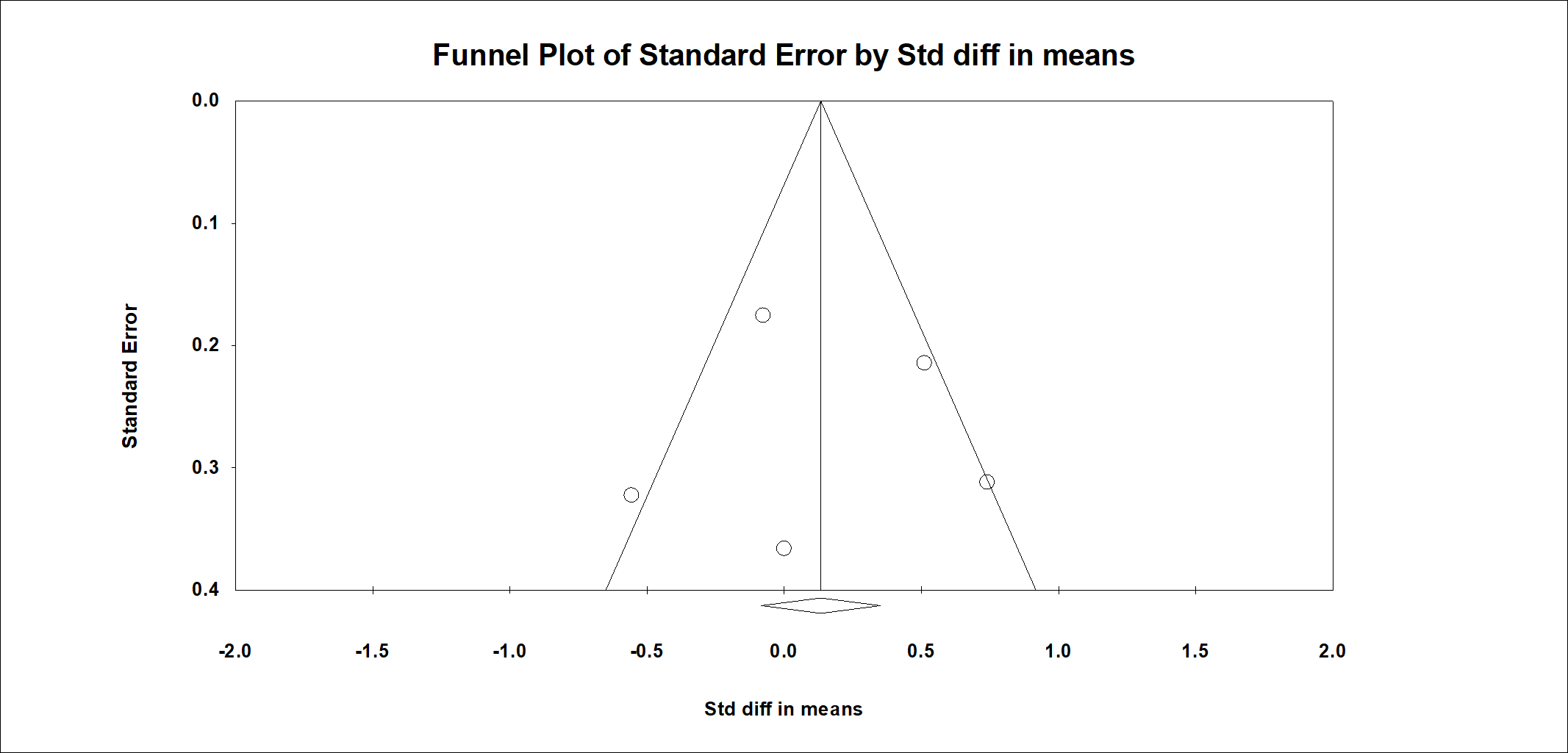

Supplement: Supplementary file 2 — Additional file 2: Figure S2. Funnel plot of Lysholm score. [file 12891_2020_3471_MOESM2_ESM.tif]

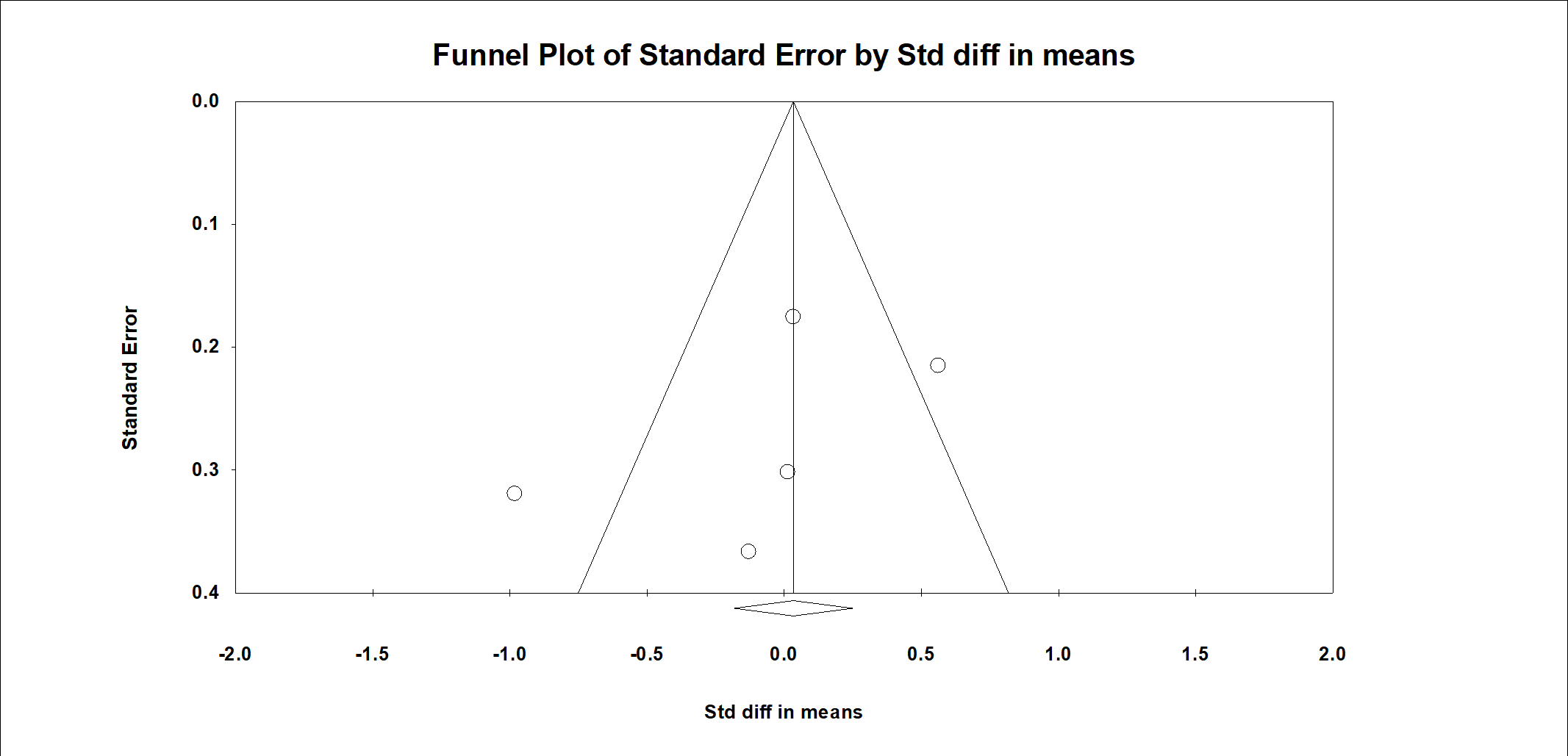

Supplement: Supplementary file 3 — Additional file 3: Figure S3. Funnel plot of subjective IKDC score. [file 12891_2020_3471_MOESM3_ESM.tif]

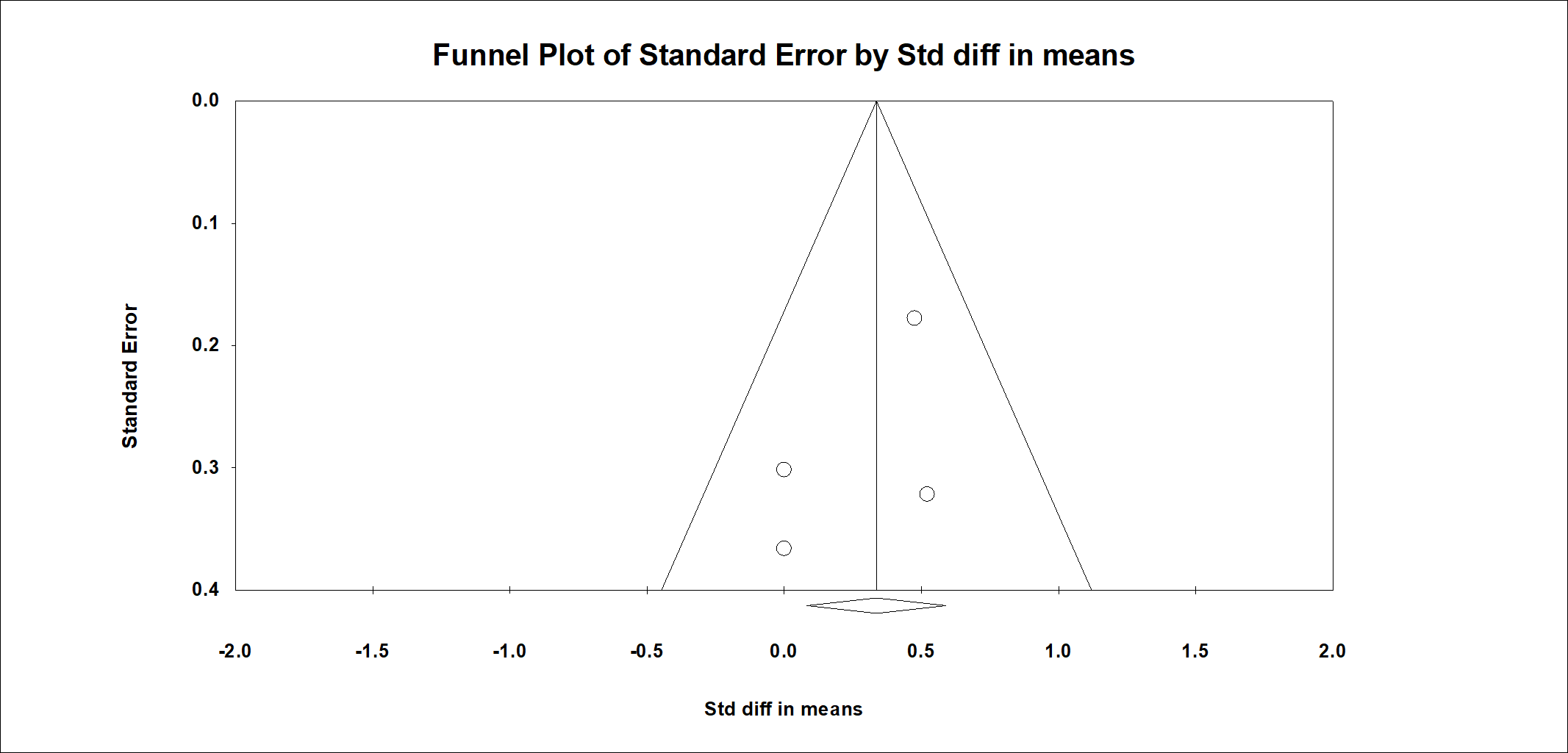

Supplement: Supplementary file 4 — Additional file 4: Figure S4. Funnel plot of Tegner score. [file 12891_2020_3471_MOESM4_ESM.tif]

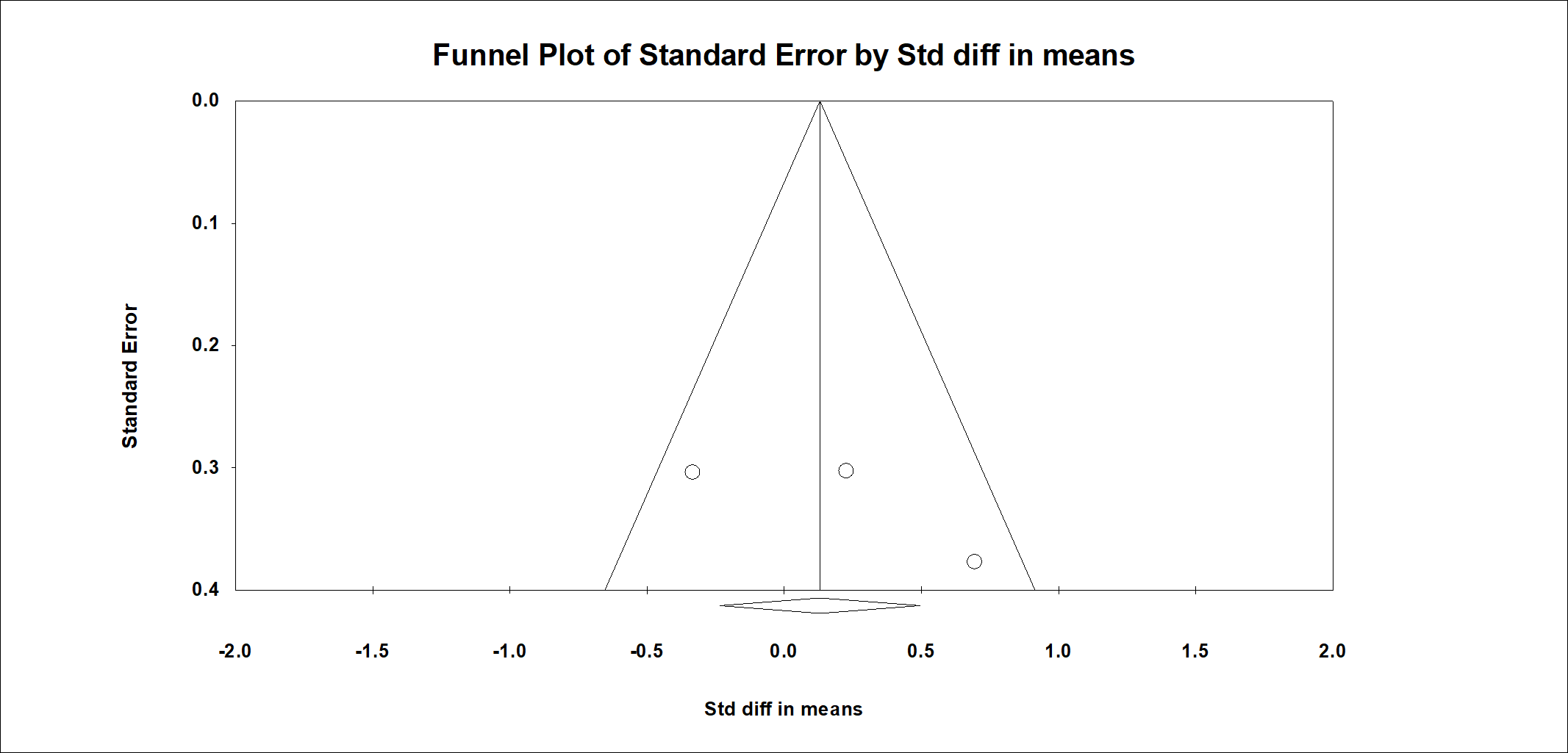

Supplement: Supplementary file 5 — Additional file 5: Figure S5. Funnel plot of the knee laxity measured by arthrometer. [file 12891_2020_3471_MOESM5_ESM.tif]

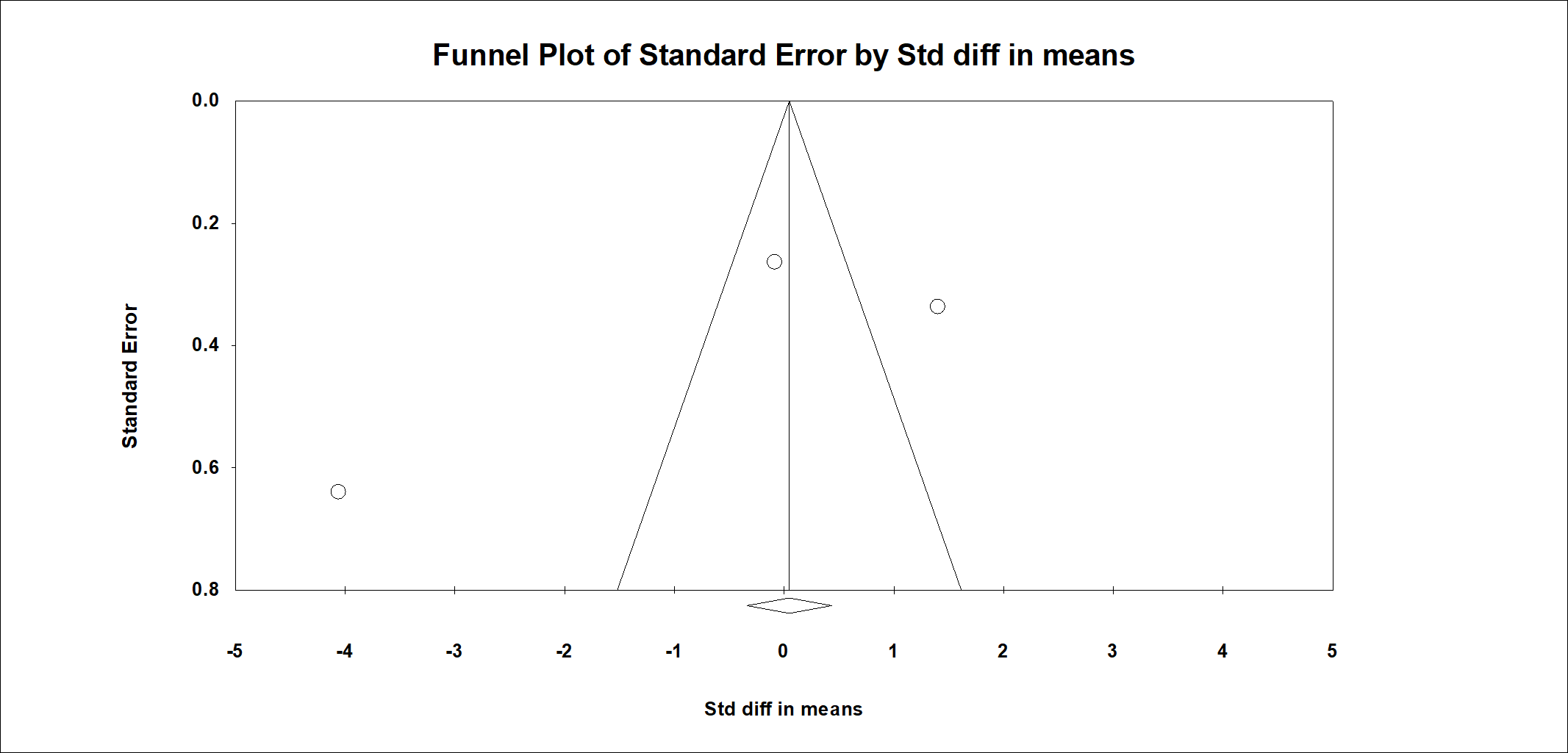

Supplement: Supplementary file 6 — Additional file 6: Figure S6. Funnel plot of the direct postoperative tunnel width. [file 12891_2020_3471_MOESM6_ESM.tif]

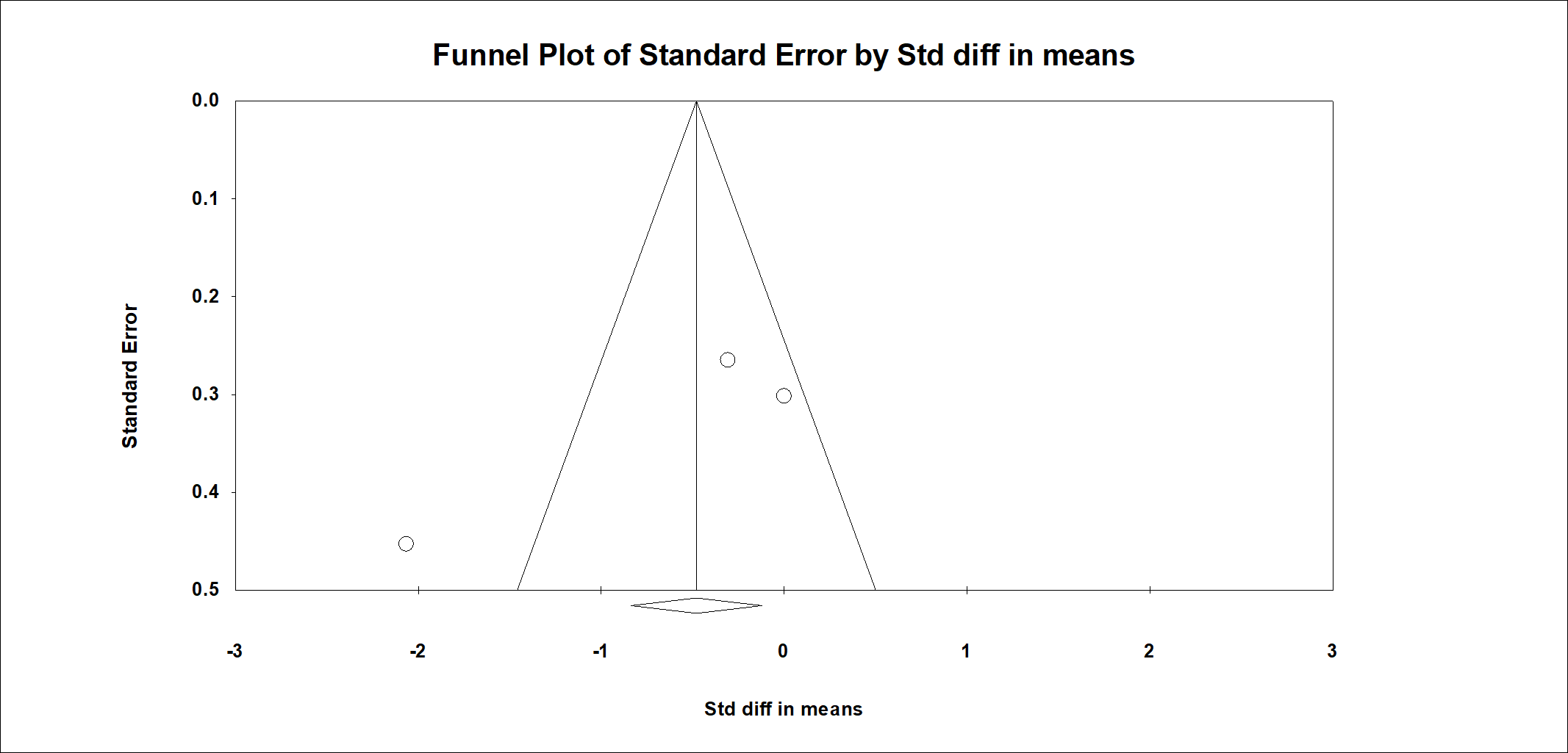

Supplement: Supplementary file 7 — Additional file 7: Figure S7. Funnel plot of the follow-up tunnel width. [file 12891_2020_3471_MOESM7_ESM.tif]

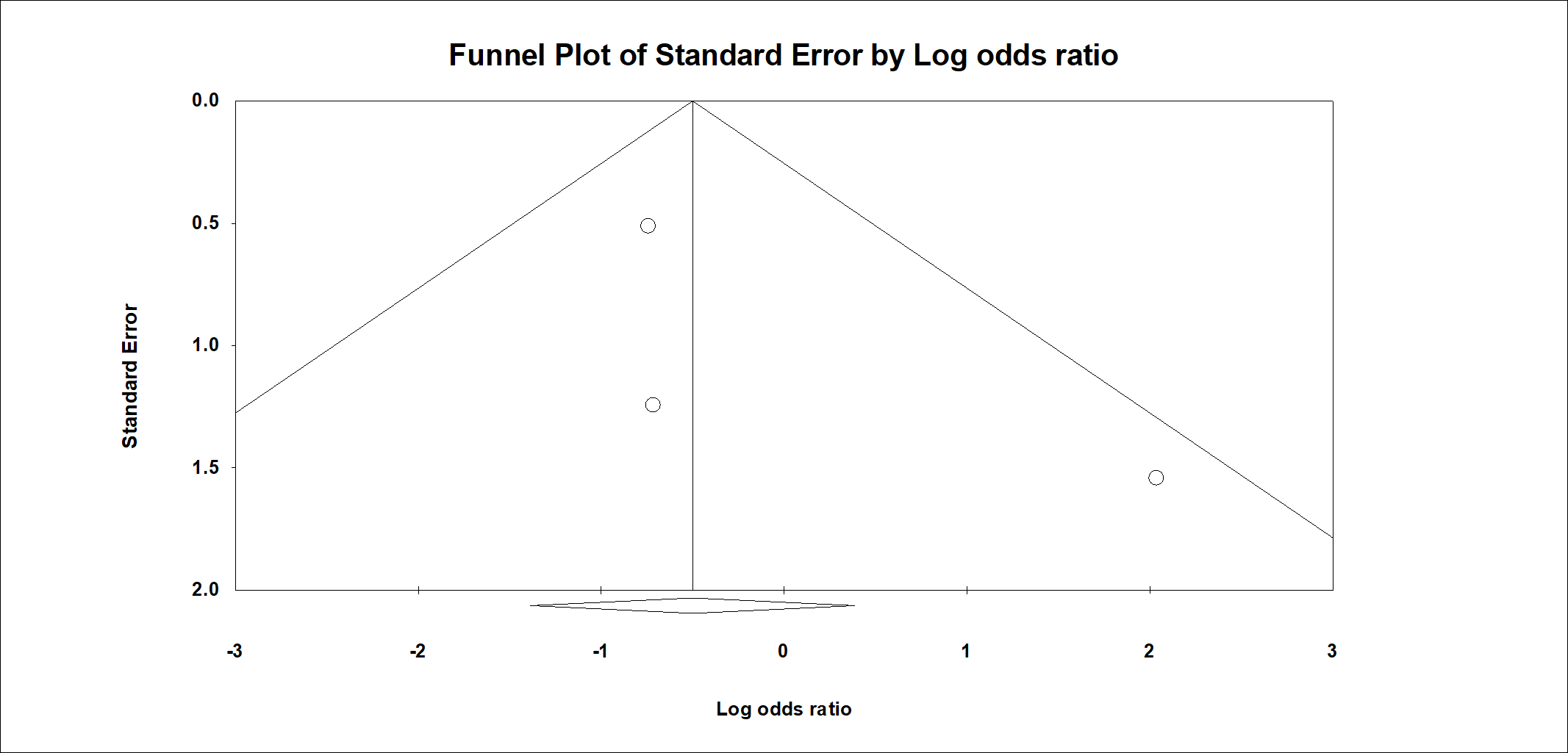

Supplement: Supplementary file 8 — Additional file 8: Figure S8. Funnel plot of the re-rupture. [file 12891_2020_3471_MOESM8_ESM.tif]
